# A Preliminary Study on Species Identification of Immature Necrophagous Phorid Flies Based on FTIR Spectroscopy

**DOI:** 10.3390/ani15213110

**Published:** 2025-10-26

**Authors:** Wutong Jia, Dianxing Feng, Yanan Tang

**Affiliations:** College of Life Science and Engineering, Shenyang University, Shenyang 110044, China; khr949110@163.com (W.J.); tyn0986@163.com (Y.T.)

**Keywords:** Fourier transform infrared spectroscopy, chemometric methods, Phoridae, indoors, immature stages

## Abstract

**Simple Summary:**

Accurate identification of the insect species is the initial step in forensic entomology case analysis. Phorid flies are significant forensic insects found on indoor and buried corpses. Due to their minute size, species identification of their immature stages presents certain challenges. This study utilized Fourier transform infrared (FTIR) combined with multivariate analytical methods to identify three common species of necrophagous phorid fly immatures. The research demonstrated that using spectral data from the fingerprint region (1800–900 cm^−1^) coupled with partial least squares-discriminant analysis (PLS-DA) effectively discriminates the species. This study provides an effective tool for the rapid identification of immature species in forensic entomology.

**Abstract:**

Phorid flies serve as the main colonizers of human remains in both indoor and burial environments. Their developmental patterns can be utilized to estimate the minimum postmortem interval (minPMI). Accurate species identification, particularly for immature stages, is essential before utilizing their developmental data to estimate minPMI. This study employed Fourier transform infrared spectroscopy (FTIR) coupled with principal components analysis (PCA) and partial least squares-discriminant analysis (PLS-DA) to investigate species identification of eggs (0 h, 8 h, 16 h), larvae (12 h, 60 h, 84 h), and pupae (1 d, 5 d, 10 d) of three necrophagous Phoridae species, *Dohrniphora cornuta*, *Diplonevra funebris*, and *Megaselia scalaris* at 24 °C. The results showed that the FTIR spectra within the fingerprint region (1800–900 cm^−1^) differed among the three immature phorid flies. These differences were primarily manifested in absorption peak intensities. The PLS-DA analysis successfully distinguished the three species at the same developmental stage. This study demonstrated the feasibility of utilizing FTIR spectroscopy coupled with chemometric methods to both rapidly identify the species of immature small flies and simultaneously estimate their age.

## 1. Introduction

Insects are the most numerous and diverse organisms on Earth [1]. They exhibit a variety of feeding habits, typically categorized as phytophagous, saprophagous, carnivorous, and omnivorous [2]. When a corpse is present, saprophagous insects, particularly necrophagous flies (e.g., blow flies and flesh flies), are among the first to arrive. They lay eggs or first-instar larvae on the corpse, whose larvae feed on it and develop. Consequently, by analyzing the developmental data of these necrophagous flies, the minimum postmortem interval (minPMI)—defined as the time between the first insect colonization of a body and the discovery of the corpse—can be estimated indirectly [3,4,5]. However, the developmental parameters, including development time [6,7,8,9], larval body length [10,11,12], intra-puparial morphological changes [13,14,15], gene expression patterns, etc. [16,17], vary significantly among different insect species. Simply applying developmental data from one species to another can lead to significant errors in minPMI estimation. Therefore, accurate species identification, particularly for immature stages, is essential before utilizing insect developmental data to estimate minPMI.

In medico-legal entomology, species identification is typically conducted by rearing larvae and pupae collected from crime scenes to adulthood and then identifying them based on morphological characteristics [1,18,19]. Alternatively, DNA-based methods, such as species identification through sequencing and alignment of the COI gene [20,21,22] or the complete mitochondrial genome [23,24,25] of juvenile insects, are relatively time-consuming and costly. In recent years, it has also been reported that gas chromatography-mass spectrometry (GC-MS) may be used for the detection of hydrocarbons in the cuticle of juvenile insects for the identification of species [26,27], but this method requires more expensive equipment.

Fourier transform infrared spectroscopy (FTIR) is a faster and cheaper technique to identify species by the position and intensity of characteristic peaks. FTIR spectroscopy demonstrates significant application value in taxonomic research. Within archeology, FTIR analysis of variations in organic matter, mineral composition, and crystallinity within human bones enables the differentiation of skeletal samples from diverse origins [28,29]. In plant taxonomy, FTIR spectroscopy not only accurately distinguishes between different plant species but also complements traditional morphological classification, thereby enhancing identification efficiency and accuracy [30]. For microbial classification, FTIR technology has been proven to be a highly efficient and rapid tool for the robust discrimination of distinct strains [31]. Similarly, in the field of forensic entomology, FTIR spectroscopy holds considerable potential for the identification of necrophagous insect species. Barbosa et al. reported that adults of *Oxysarcodexia timida* (Aldrich, 1916), *O. thornax* (Walker, 1849), *Peckia chrysostoma* (Wiedemann, 1830), *P. lambens* (Wiedemann, 1830), *Ravinia belforti* (Lopes, 1939), and *Tricharaea occidua* (Wiedemann, 1830) (Diptera: Sarcophagidae) native to Neotropical regions could be distinguished by Attenuated total reflection Fourier transform infrared (ATR-FTIR) spectroscopy combined with variable selection techniques such as genetic algorithm-linear discriminant analysis (GA-LDA) and successive projection algorithm-linear discriminant analysis (SPA-LDA) [32]. Pickering et al. utilized ART-FTIR spectroscopy coupled with a support vector machine (SVM) algorithm to rapidly discriminate the maggots of *Calliphora vomitoria* Linnaeus, 1758, *Lucilia sericata* (Meigen, 1826) (Diptera: Calliphoridae), and *Musca domestica* Linnaeus, 1758 (Diptera: Muscidae), with a cross-validation accuracy of 86.21% [33]. Zhang et al. employed ATR-FTIR spectroscopy to acquire spectral data from empty pupariums of five fly species and combined this with machine learning methods, including SVM, artificial neural networks (ANN), and random forest (RF), for species differentiation, with the SVM model demonstrating the highest classification accuracy [34].

Compared to larger fly species, diminutive phorid flies can penetrate enclosures that are relatively sealed (such as closed rooms, vehicle interiors, sealed plastic bags, and coffins) through narrow crevices [35,36,37,38,39]. They are capable of rapidly colonizing corpses and may become the primary or even the only entomological evidence available at crime scenes [40]. This study aims to systematically evaluate the effectiveness of FTIR spectroscopy for species identification of phorid flies at immature developmental stages (egg, larva, and pupa), thereby establishing a rapid diagnostic protocol based on spectral signatures to facilitate accurate species determination of immature phorid flies in forensic entomology practice.

## 2. Materials and Methods

### 2.1. Insect

The adult specimens of *Dohrniphora cornuta* (Bigot, 1857), *Diplonevra funebris* (Meigen, 1826), and *Megaselia scalaris* (Loew, 1866) were collected using rotten lean pork as bait in Shenyang City (123°25′ E, 41°48′ N), Liaoning Province, China. Each specimen was identified following the taxonomic keys [41]. All species were reared in the laboratory on fresh lean pork and maintained at 21–24 °C and 75% relative humidity with a 12 h light-dark photoperiod [42].

### 2.2. Sample Preparation

For each of the three species, 3–5 pairs of adult flies were introduced into a 1000 mL narrow-necked bottle (Sichuan Shubo Co., Ltd., Chongzhou, China). Fresh lean pork was placed inside the bottle to attract oviposition. The bottle was sealed with industrial filter cloth (Suzhou Tebang Environmental Protection Technology Co., Ltd., Suzhou, China). The bottle was then placed in an artificial climate chamber (Ningbo Laifu Technology Co., Ltd., Ningbo, China) set at 24 °C, 75% relative humidity, and a 12 h light-dark photoperiod. Upon detection of eggs, timing was initiated; eggs laid within 2 h were designated as the 0 h eggs. These eggs were gently transferred using a soft, fine-bristle brush into Petri dishes. An appropriate amount of distilled water was sprayed to maintain humidity, and the dishes were sealed with parafilm. Timing for larval development commenced upon the emergence of the first-instar larva; first-instar larvae hatched within 2 h were designated as 0 h larvae. The larvae were then gently transferred into 1000 mL narrow-necked bottles containing fresh lean pork as a food source. Larval growth was monitored daily. Timing for pupal development commenced upon observation of the first prepupa formation. The prepupae formed within 2 h were designated as 0 h pupae. The pupae were transferred into Petri dishes, which were then sealed with parafilm for daily observations. Throughout the development of the three phorid fly species, samples were collected as follows: eggs at 0 h, 8 h, and 16 h (nine eggs per time point); larvae at 12 h, 60 h, and 84 h (three larvae per time point); and pupae at 1 d, 5 d, and 10 d (three pupae per time point). The selected time points represent the early, middle, and late developmental stages of eggs, larvae, and pupae. Collected samples were frozen and stored at −80 °C.

### 2.3. Collection of FTIR Spectra

The samples were thoroughly rinsed with distilled water before using. For egg stage sample preparation, three eggs were pooled as one independent sample and ground thoroughly in an agate mortar with 0.02 g of spectroscopic-grade KBr (Shanghai Macklin Biochemical Co., Ltd., Shanghai, China). The entire homogenate was pressed into a transparent pellet in the manual hydraulic press (Specac Co., Ltd., Kent, UK). For larval and pupal stages, an individual larva or pupa was weighed using an analytical balance (Tianma Balance Instrument Co., Ltd., Tianjin, China). Spectroscopic-grade KBr was then weighed according to a 1:100 (*w*/*w*) ratio (larva/pupa:KBr). The specimen and KBr were combined in an agate mortar and ground thoroughly. Subsequently, 0.02 g of the mixture was accurately weighed and then transferred into a pellet die set, subsequently compressed into a transparent pellet.

The prepared pellet was mounted on the sample stage of an Agilent Cary 630 FTIR spectrometer (Agilent Technologies, Inc., Santa Clara, CA, USA) for spectral acquisition. Spectra were collected over the range of 4000–400 cm^−1^ with 32 scans per measurement and a resolution of 4 cm^−1^. Technical triplicates were performed for each sample. For each species at each time point, three independent biological replicates were analyzed, resulting in the use of a total of 81 eggs, 27 larvae, and 27 pupae. A background spectrum was collected immediately before each sample measurement. After completing data collection for each sample, the pellet die set was meticulously cleaned with distilled water, followed by absolute ethanol to avoid cross-contamination.

### 2.4. Data Analysis

The intensities of the major absorption peaks in the biological fingerprint region across different developmental stages of three phorid fly species were compared using the Kruskal–Wallis test, followed by Dunn’s post hoc test for pairwise comparisons. Statistical significance was defined as α = 0.05. All statistical analyses were performed using GraphPad Prism 9.5 software.

The raw spectral data were subjected to first-order derivative transformation using the Savitzky–Golay convolution algorithm (15 smoothing points) in OMNIC 9.2 software, followed by standard normal variate (SNV) transformation performed in MATLAB R2024a. The preprocessed data were split into training and test sets at a ratio of 7:3. Based on the training set, principal component analysis (PCA) and partial least squares-discriminant analysis (PLS-DA) models were established in SIMCA 14.1. The optimal model was selected through comparative analysis of model parameters and visualization results. The performance of the chosen model was ultimately evaluated using the test set. A confusion matrix was generated with R 4.4.3 software.

## 3. Results

### 3.1. FTIR Spectra of Three Necrophagous Phorid Flies at Different Developmental Stages

**Figure 1:**
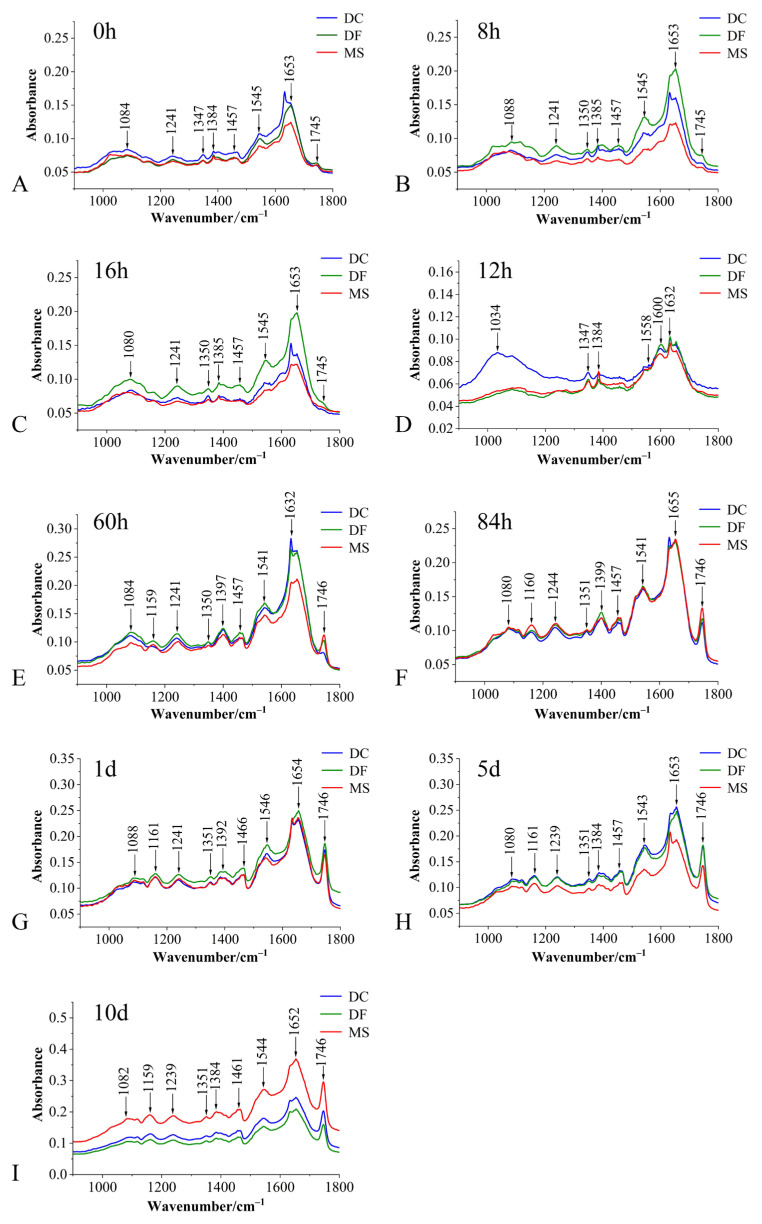
Average spectra of the biological fingerprint region for three Phoridae species at different developmental stages. (**A**–**C**): egg stage; (**D**–**F**): larval stage; (**G**–**I**): pupal stage; DC: *Dohrniphora cornuta*; DF: *Diplonevra funebris*; MS: *Megaselia scalaris*.

**Figure 2:**
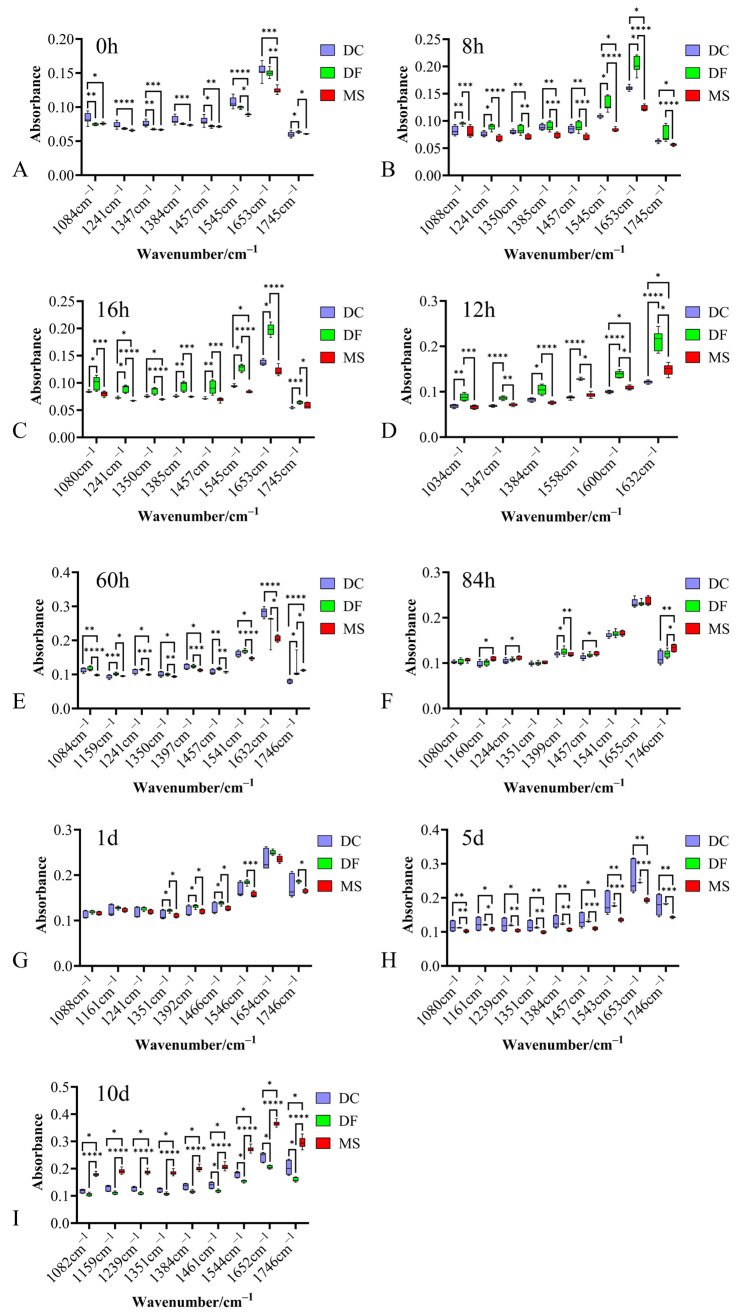
Box plot of absorption peak intensities in the biological fingerprint region spectra for three Phoridae species at different developmental stages. (**A**–**C**): egg stage; (**D**–**F**): larval stage; (**G**–**I**): pupal stage; DC: *Dohrniphora cornuta*; DF: *Diplonevra funebris*; MS: *Megaselia scalaris*. * represents *p* < 0.05, ** represents *p* < 0.01, *** represents *p* < 0.001, **** represents *p* < 0.0001.

The average spectra of the fingerprint region for the three phorid fly species at immature stages are shown in Figure 1. Eggs of all three species exhibited intense absorption peaks near 1084 cm^−1^, 1241 cm^−1^, 1350 cm^−1^, 1384 cm^−1^, 1455 cm^−1^, 1544 cm^−1^, 1645 cm^−1^, and 1746 cm^−1^ (Figure 1). While no species-specific characteristic absorption peaks were identified, significant differences in peak intensities were observed among the species (Figure 1 and Figure 2).

Compared to the average spectra of eggs and 12 h larvae, distinct differences in absorbance at the 1159 cm^−1^ peak were also evident in 60 h and 84 h larvae, and pupae (Figure 1A–I).

These absorption peaks primarily reflect vibrational characteristics of functional groups or chemical bonds associated with carbohydrates, lipids, proteins, and DNA within the tissues (Table 1).

### 3.2. Discrimination Analysis of Three Necrophagous Phorid Flies at the Identical Developmental Time Points

Based on the FTIR data from the biological fingerprint region, PCA and PLS-DA models were established for each immature stage at 24 °C.

**Figure 3:**
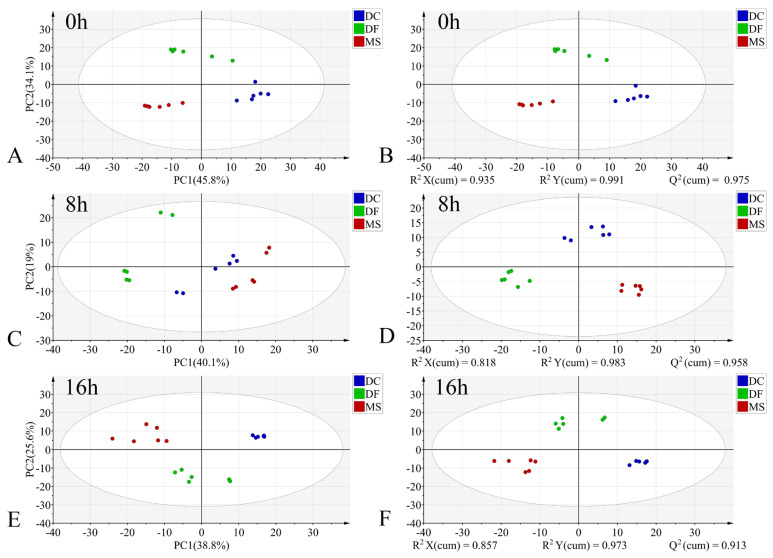
PCA and PLS-DA score plots of FTIR data in the biological fingerprint region for three Phoridae species at the same time point during the egg stage. (**A**,**C**,**E**): PCA; (**B**,**D**,**F**): PLS-DA; DC: *Dohrniphora cornuta*; DF: *Diplonevra funebris*; MS: *Megaselia scalaris*.

**Figure 4:**
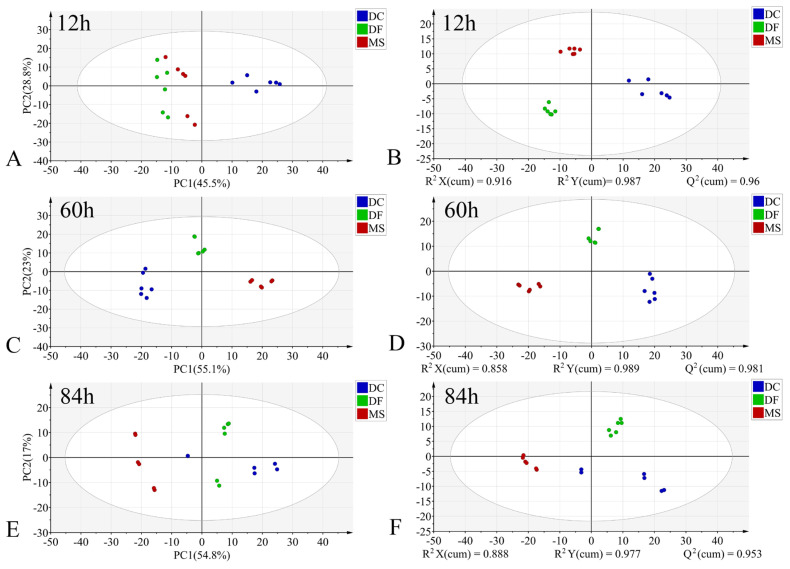
PCA and PLS-DA score plots of FTIR data in the biological fingerprint region for three Phoridae species at the same time point during the larval stage. (**A**,**C**,**E**): PCA; (**B**,**D**,**F**): PLS-DA; DC: *Dohrniphora cornuta*; DF: *Diplonevra funebris*; MS: *Megaselia scalaris*.

**Figure 5:**
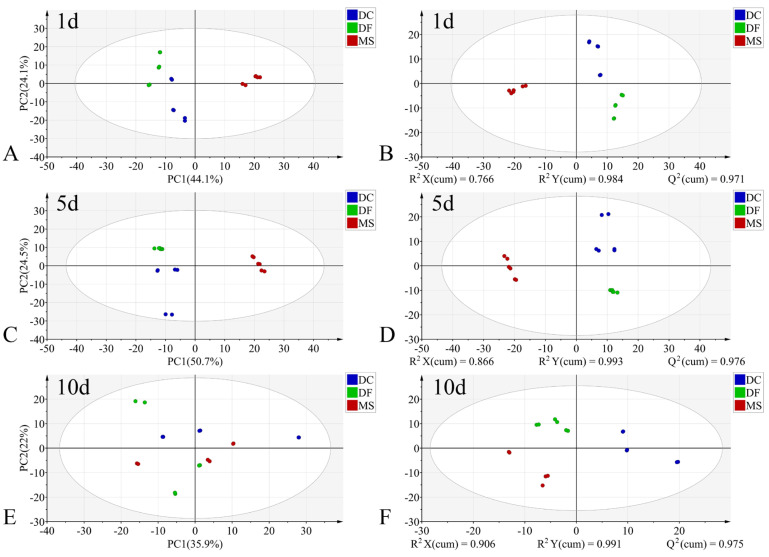
PCA and PLS-DA score plots of FTIR data in the biological fingerprint region for three Phoridae species at the same time point during the pupal stage. (**A**,**C**,**E**): PCA; (**B**,**D**,**F**): PLS-DA; DC: *Dohrniphora cornuta*; DF: *Diplonevra funebris*; MS: *Megaselia scalaris*.

In the PCA models, the first two principal components (PC1 and PC2) collectively accounted for the following proportions of variance in the original data: 59.1–79.9% for the egg stage, 71.8–78.1% for the larval stage, and 57.9–75.2% for the pupal stage (Figure 3A,C,E, Figure 4A,C,E and Figure 5A,C,E). Samples from different species showed partial clustering by species; however, the sample points were relatively scattered with overlapping clusters (Figure 4A,E and Figure 5E).

In the PLS-DA models, samples at identical time points exhibited clearer clustering trends compared to the PCA models (Figure 3B,D,F, Figure 4B,D,F and Figure 5B,D,F). The model parameters indicated good performance: R^2^X > 0.766, R^2^Y > 0.973, and Q^2^ > 0.913, reflecting strong explanatory and predictive capabilities. Permutation tests further confirmed the models were not overfitted (R^2^ < 0.391, Q^2^ < −0.368). External validation using the test set demonstrated 100% classification accuracy for all PLS-DA models (Table 2).

### 3.3. Discrimination Analysis of Three Necrophagous Phorid Flies at the Same Developmental Stage

In addition to the biological fingerprint region (1800–900 cm^−1^) spectral data, we also constructed PLS-DA models using the full-range spectra (4000–400 cm^−1^), VIP-selected full-range features (4000–400 cm^−1^, VIP > 1), and VIP-selected fingerprint region features (1800–900 cm^−1^, VIP > 1) to analyze and compare sample clustering patterns within the same developmental stage.

**Figure 6:**
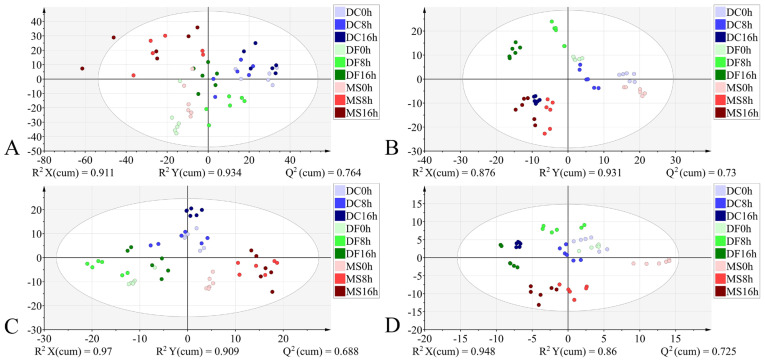
PLS-DA score plots based on the different datasets from the egg stage. (**A**): 4000–400 cm^−1^; (**B**): 4000–400 cm^−1^ (VIP > 1); (**C**): 1800–900 cm^−1^; (**D**): 1800–900 cm^−1^ (VIP > 1). DC: *Dohrniphora cornuta*; DF: *Diplonevra funebris*; MS: *Megaselia scalaris*.

**Figure 7:**
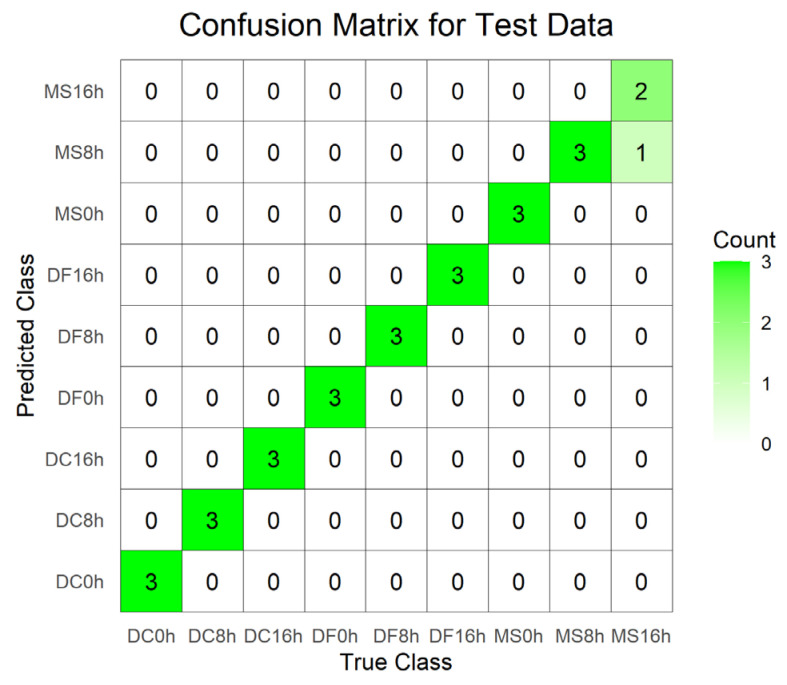
Confusion matrix of the test set for the PLS-DA model constructed from spectral data 4000–400 cm^−1^ (VIP > 1) of the egg stage. DC: *Dohrniphora cornuta*; DF: *Diplonevra funebris*; MS: *Megaselia scalaris*.

For the egg stage, the PLS-DA model constructed using the 1800–900 cm^−1^ dataset exhibited good performance: R^2^X = 0.97, R^2^Y = 0.909, Q^2^ = 0.688, the model has a good degree of interpretation and prediction, permutation test intercepts R^2^ = 0.262 and Q^2^ = −1.17, without fitting, the accuracy of model evaluation is 100% (Table 3). In the corresponding score plot (Figure 6C), samples from different developmental times clustered by species. In contrast, the discriminant model built using the full-range 4000–400 cm^−1^ dataset showed signs of overfitting (permutation test R^2^ = 0.416, Q^2^ = −1.39) (Table 3). Its score plot revealed high dispersion of sample points across different species and developmental times, with indistinct clustering trends (Figure 6A). The model utilizing the 4000–400 cm^−1^ (VIP > 1) dataset achieved a classification accuracy of 96.3% (Table 3), but incurred one misclassification where a 16 h *M. scalaris* egg was incorrectly assigned to the 8 h group (Figure 7). Conversely, the discriminant model based on 1800–900 cm^−1^ (VIP > 1) demonstrated robust performance: R^2^X = 0.948, R^2^Y = 0.86, Q^2^ = 0.725, permutation test intercepts R^2^ = 0.198 and Q^2^ = −1.05, confirming good explanatory and predictive power, no overfitting, and 100% classification accuracy (Table 3). Its score plot (Figure 6D) showed reduced sample dispersion and clearer clustering. Notably, along the *X*-axis, samples clustered primarily according to developmental time within each species.

**Figure 8:**
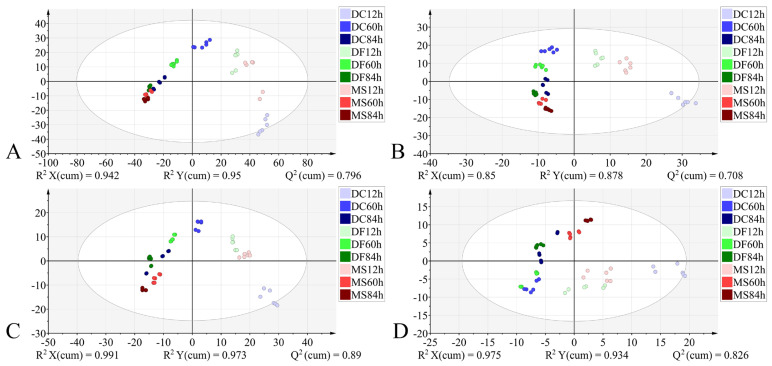
PLS-DA score plots based on the different datasets from the larval stage. (**A**): 4000–400 cm^−1^; (**B**): 4000–400 cm^−1^ (VIP > 1); (**C**): 1800–900 cm^−1^; (**D**): 1800–900 cm^−1^ (VIP > 1). DC: *Dohrniphora cornuta*; DF: *Diplonevra funebris*; MS: *Megaselia scalaris*.

During the larval stage, models constructed using all four datasets exhibited favorable classification performance: R^2^X > 0.85, R^2^Y > 0.878, Q^2^ > 0.708, demonstrating good model fit. External validation achieved 100% accuracy. However, permutation testing revealed overfitting in the model based on the 4000–400 cm^−1^ dataset (Table 4). The model utilizing the 1800–900 cm^−1^ dataset outperformed others in terms of explanatory power, predictive capability, and visual clustering in the score plot. Samples from the three phorid fly species exhibited a stronger tendency to cluster by developmental time than by species along the *X*-axis (Figure 8, Table 4).

**Figure 9:**
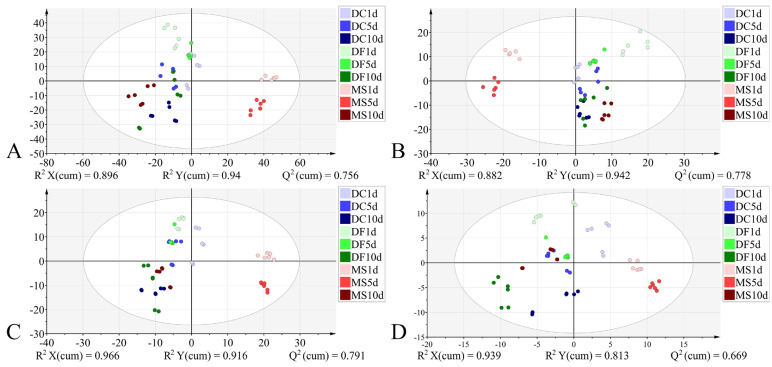
PLS-DA score plots based on the different datasets from the pupal stage. (**A**): 4000–400 cm^−1^; (**B**): 4000–400 cm^−1^ (VIP > 1); (**C**): 1800–900 cm^−1^; (**D**): 1800–900 cm^−1^ (VIP > 1). DC: *Dohrniphora cornuta*; DF: *Diplonevra funebris*; MS: *Megaselia scalaris*.

For the pupal stage, models constructed using all four datasets demonstrated good performance: R^2^X > 0.882, R^2^Y > 0.813, Q^2^ > 0.669, indicating strong explanatory and predictive capabilities, with all achieving 100% classification accuracy. However, permutation tests indicated the presence of overfitting in models constructed using both the full 4000–400 cm^−1^ spectral range and the VIP-filtered (VIP > 1) subset of this range (Table 5). The model based on the 1800–900 cm^−1^ dataset demonstrated superior interpretability and predictive capability compared to the model using VIP > 1 from the same spectral range (Table 5). In the corresponding score plot (Figure 9), samples generally exhibited distinct species-specific clustering at identical developmental times. Notably, along the *Y*-axis, samples from the three phorid species showed a trend of grouping by developmental time.

## 4. Discussion

FTIR spectroscopy enables rapid analysis of trace biological samples by detecting vibrational signatures of chemical groups within biomolecules, directly reflecting molecular structure and composition. While widely reported for species identification in plants and mammals [43,44,45], its application in forensic entomology remains limited. To date, studies have only demonstrated its use for discriminating six species of neotropical Sarcophagidae adults [32], larvae of *C. vomitoria*, *L. sericata*, and *M. domestica* [33], as well as empty pupariums of *Aldrichina grahami*, *Sarcophaga africa*, *Chrysomya megacephala*, *Sarcophaga peregrina*, and *M. domestica* [34]. This study employed FTIR spectroscopy to detect changes in chemical functional groups during the immature stages of necrophagous phorid flies for the first time.

Distinct differences were observed in the average FTIR spectra of eggs (0 h, 8 h, 16 h), larvae (12 h, 60 h, 84 h), and pupae (1 d, 5 d, 10 d) across three phorid fly species: *D. cornuta*, *D. funebris*, and *M. scalaris*. PCA and PLS-DA are two important multivariate analysis methods in chemometrics, with PCA being an unsupervised pattern recognition method and PLS-DA being a supervised one [46]. Two distinct models, the PCA and PLS-DA, were constructed based on the spectral data in this study. In most PCA models, the cumulative variance captured by the first two components is below 75%, which is considered suboptimal. Compared to the PCA model, the PLS-DA model demonstrated tighter clustering of samples belonging to the same species in the score plot, indicating superior performance for this purpose.

Subsequently, we established PLS-DA models for three different developmental stages of phorid flies using four different datasets. For the egg stage, the model constructed using the 4000–400 cm^−1^ dataset exhibited the highest R^2^Y (0.934) and Q^2^ (0.764) values among all datasets. However, permutation validation revealed R^2^ > 0.4, indicating overfitting, and the score plot showed high dispersion among sample points. The model built with the 4000–400 cm^−1^ (VIP > 1) dataset resulted in misclassification. When using the 1800–900 cm^−1^ dataset and the 1800–900 cm^−1^ (VIP > 1) dataset, the former model demonstrated superior parameters except for a slightly lower Q^2^ compared to the latter. Permutation tests confirmed neither model was overfitted. Sample points in the score plot of the 1800–900 cm^−1^ model exhibited lower dispersion and more distinct clustering. For the larval stage, the model based on the 4000–400 cm^−1^ dataset showed overfitting. The other three datasets did not exhibit overfitting. Among these, the 1800–900 cm^−1^ dataset yielded optimal model parameters and the lowest dispersion of sample points in the score plot. For the pupal stage, models built with both the 4000–400 cm^−1^ and 4000–400 cm^−1^ (VIP > 1) datasets displayed overfitting. The model constructed using the 1800–900 cm^−1^ dataset demonstrated better parameters than the model based on the 1800–900 cm^−1^ (VIP > 1) dataset. Sample points in the score plot of the 1800–900 cm^−1^ model showed lower dispersion. In summary, after comparing model parameters and score plots, we conclude that the model constructed using the 1800–900 cm^−1^ spectral data is optimal. This is consistent with the findings of previous studies [32,47,48,49,50].

Furthermore, in the score plots generated using the 1800–900 cm^−1^ dataset, besides the distinct clustering of egg samples by species at different developmental time points, larval and pupal stage samples also exhibited clustering according to developmental time. In the score plots generated using the 1800–900 cm^−1^ (VIP > 1) dataset, samples from the egg, larval, and pupal stages all exhibited a trend of clustering by developmental time, particularly evident during the early stage of each developmental period. Recently, some researchers also reported that it is feasible to determine the intra-puparial age of *Calliphora vicina* and *Phormia regina* by combining FTIR spectroscopy with chemometric methods [46,51].

This indicates that FTIR spectroscopy combined with chemometric methods is applicable not only for identifying species during the immature stages of phorid flies, but also for determining their age. In forensic entomology practice, larval or pupal specimens collected at crime scenes are typically subjected to species identification via morphological or molecular biological methods [1,2]. Once the species is determined, the minPMI is subsequently estimated based on the species-specific developmental data. In contrast, FTIR requires only a single set of spectral data to simultaneously achieve species identification and developmental stage determination within a few hours. This dual-purpose analytical capability provides a novel technological pathway for the rapid and efficient estimation of the minPMI.

However, this study is preliminary and has several limitations. Sampling was conducted at only three time points per developmental stage (early, middle, and late), with a limited number of specimens. It remains unclear whether similar discriminatory performance could be achieved with larger sample sizes and higher sampling density. Furthermore, the effectiveness of this method in identifying larvae of different instars requires further investigation. Samples in this study were directly frozen and stored at −80 °C. In forensic entomology, however, a common preservation method involves heat or Carnoy’s solution killing larvae or pupae, followed by immersion in ethanol [52,53,54]. Whether samples preserved using this method exhibit FTIR spectral differences compared to frozen samples is uncertain, given that heating, ethanol, and Carnoy’s solution can break hydrogen bonds and ionic bonds in nucleic acids and protein conformations, thereby denaturing these macromolecules [55,56]. Additionally, different geographical environments can also lead to variations in the FTIR spectra of species, such as *Dendrobium officinale* [57] and *Larimichthys crocea* [58], thereby affecting species identification results based on infrared spectroscopy. Nevertheless, our study did not account for this factor. There is no doubt that the combination of morphological and molecular techniques currently represents the most reliable approach for insect identification. The FTIR method cannot yet replace them. However, FTIR technology demonstrates unique advantages in estimating minPMI, as it enables simultaneous species identification and age determination. This positions FTIR as a promising tool for future applications. Nevertheless, before this method can be practically implemented, substantial work remains to be done. This includes validating its effectiveness on specimens preserved in organic solvents and dried specimens, evaluating its performance in distinguishing different instars larvae and geographic populations, and establishing an FTIR spectral database for the immature stages of common necrophagous flies.

## 5. Conclusions

This study represents the first analysis of the FTIR spectral characteristics of three species of immature phorid flies. Distinct differences were observed in the intensity of the major absorption peaks within the fingerprint region (1800–900 cm^−1^) among the three Phoridae species. The classification performance of PLS-DA was superior to that of PCA. The FTIR combined with PLS-DA not only effectively discriminated phorid flies at the same developmental stage, but also simultaneously estimated their age. Our work demonstrates the significant application potential of FTIR spectroscopy coupled with multivariate analysis techniques for the rapid identification of immature stages of necrophagous insect species.

## Figures and Tables

**Table 1:** Identification of the main absorption peaks in the fingerprint region of three Phoridae species.

Wavenumbers/cm^−1^	Infrared Band Assignment
1100–1000	C-O asymmetric stretching vibration or $PO_{2}^{-}$ symmetric stretching vibration: carbohydrates/DNA
1180–1134	C-O (H) stretching vibration: lipid
1260–1180	Amide III C-O bending vibration or C-N stretching vibration: protein
1360–1336	C-H_2_ rocking vibration
1420–1370	COO^−^ symmetric stretching vibration: lipids/amino acids
1480–1430	C-H asymmetric bending vibration: lipids
1580–1510	Amide II C-N stretching vibration or N-H bending vibration: protein
1680–1600	Amide I C=O stretching vibration: protein
1760–1730	C=O stretching vibration: lipid

**Table 2:** Cross-validation, permutation test, and external validation of the PLS-DA models for different developmental stages of three phorid flies.

Developmental Stages	Developmental Time	Cross-Validation			Permutation Test		External Validation
		R^2^X (cum)	R^2^Y (cum)	Q^2^ (cum)	R^2^	Q^2^	Accuracy
Egg	0 h	0.935	0.991	0.975	0.265	−0.508	100%
	8 h	0.818	0.983	0.958	0.345	−0.613	100%
	16 h	0.857	0.973	0.913	0.324	−0.55	100%
Larva	12 h	0.916	0.987	0.96	0.304	−0.539	100%
	60 h	0.858	0.989	0.981	0.236	−0.368	100%
	84 h	0.888	0.977	0.953	0.313	−0.584	100%
Pupa	1 d	0.766	0.984	0.971	0.241	−0.447	100%
	5 d	0.866	0.993	0.976	0.324	−0.49	100%
	10 d	0.906	0.991	0.975	0.391	−0.748	100%

**Table 3:** Cross-validation, permutation test, and external validation of the PLS-DA models for the egg stage across different datasets.

Dataset	Cross-Validation			Permutation Test		External Validation
	R^2^X (cum)	R^2^Y (cum)	Q^2^ (cum)	R^2^	Q^2^	Accuracy
4000–400 cm^−1^	0.911	0.934	0.764	0.416	−1.39	100%
4000–400 cm^−1^ (VIP > 1)	0.876	0.931	0.73	0.353	−1.43	96.3%
1800–900 cm^−1^	0.97	0.909	0.688	0.262	−1.17	100%
1800–900 cm^−1^ (VIP > 1)	0.948	0.86	0.725	0.198	−1.05	100%

**Table 4:** Cross-validation, permutation test, and external validation of the PLS-DA models for the larval stage across different datasets.

Dataset	Cross-Validation			Permutation Test		External Validation
	R^2^X (cum)	R^2^Y (cum)	Q^2^ (cum)	R^2^	Q^2^	Accuracy
4000–400 cm^−1^	0.942	0.95	0.796	0.489	−1.29	100%
4000–400 cm^−1^ (VIP > 1)	0.85	0.878	0.708	0.285	−0.96	100%
1800–900 cm^−1^	0.991	0.973	0.89	0.361	−1.6	100%
1800–900 cm^−1^ (VIP > 1)	0.975	0.934	0.826	0.253	−1.27	100%

**Table 5:** Cross-validation, permutation test, and external validation of the PLS-DA models for the pupal stage across different datasets.

Dataset	Cross-Validation			Permutation Test		External Validation
	R^2^X (cum)	R^2^Y (cum)	Q^2^ (cum)	R^2^	Q^2^	Accuracy
4000–400 cm^−1^	0.896	0.94	0.756	0.453	−1.39	100%
4000–400 cm^−1^ (VIP > 1)	0.882	0.942	0.778	0.424	−1.51	100%
1800–900 cm^−1^	0.966	0.916	0.791	0.284	−1.41	100%
1800–900 cm^−1^ (VIP > 1)	0.939	0.813	0.669	0.152	−0.919	100%

## Data Availability

Data are provided within the article. Further details or information can be directed to the corresponding author.
